# Intracellular iron positively modulates the replicative cycle of mimiviruses, increasing virus production

**DOI:** 10.1128/jvi.01025-25

**Published:** 2025-10-01

**Authors:** Juliana dos Santos Oliveira, Claudia F. Dick, Gabriel Henrique Pereira Nunes, Victor Alejandro Essus, Jason Schrad, Sundharraman Subramanian, Jônatas Abrahão, José Roberto Meyer-Fernandes, Kristin Parent, Juliana Reis Cortines

**Affiliations:** 1 Laboratório de Virologia e Espectrometria de Massas, Instituto de Microbiologia Paulo de Góes, Departamento de Virologia, Universidade Federal do Rio de Janeiro 28125, Rio de Janeiro, Brazil; 2 Laboratório de Bioquímica Celular, Instituto de Bioquímica Médica Leopoldo De Meis, Universidade Federal do Rio de Janeiro 28125, Rio de Janeiro, Brazil; 3 Cell Biology Department, Southwestern Medical Center, University of Texas 12334, Dallas, Texas, USA; 4 Department of Biochemistry and Molecular Biology, Michigan State University 3078, East Lansing, Michigan, USA; 5 Virus laboratory, Institute of Biological Sciences, Department of Microbiology, Federal University of Minas Gerais 28114, Belo Horizonte, Minas Gerais, Brazil; Northwestern University Feinberg School of Medicine, Chicago, Illinois, USA

**Keywords:** *Acanthamoeba polyphaga mimivirus*, Tupanvirus, stargate, iron, nutritional immunity

## Abstract

Mimiviruses, members of the *Nucleocytoviricota* phylum, produce large, pseudo-icosahedral virions densely coated with fibrils, except at a specialized fivefold vertex known as the stargate. Stargate opening is essential for delivering the viral core into the cytoplasm of phagocytic amoebae. These professional phagocytes deploy antimicrobial mechanisms such as the modulation of transition metal concentrations within phagolysosomes to eliminate internalized pathogens. Yet, mimiviruses have evolved striking adaptations to resist such hostile environments and initiate replication within these compartments. Here, we investigated the role of metal ions—particularly iron—in the replication of three giant viruses: *Acanthamoeba polyphaga mimivirus* (APMV), Antarctica virus, and Tupanvirus (TPV). We show that infection by these viruses increases cellular iron uptake and that elevated intracellular iron enhances viral replication. These findings reveal a previously underappreciated facet of the mimivirus–host interaction, in which iron availability acts as a positive modulator of the viral replicative cycle.

**IMPORTANCE:**

Giant viruses like Mimivirus infect amoebae, which normally destroy microbes using toxic conditions inside cellular compartments. This study shows that, instead of being harmed, these viruses benefit from one of those supposedly hostile factors: iron. Through this work, we discovered that infection by Mimivirus and related viruses increases the host cell’s iron uptake—and that more iron boosts virus production by the host cell. This reveals a surprising twist in the virus–host relationship: what should be a defense mechanism is turned into an advantage by the virus. By highlighting iron as a key factor in viral success, this work opens new perspectives on how giant viruses adapt to—and even exploit—the internal environment of their hosts. It also adds an important piece to our understanding of the complex strategies viruses use to survive and thrive inside cells.

## INTRODUCTION

Professional phagocytic cells refer to those specialized in phagocytizing foreign bodies, including free-living organisms, like amoebas, macrophages, leucocytes, and neutrophils (1). They utilize phagocytosis to feed (amoebas) or to maintain the organism’s homeostasis (macrophages, leucocytes, and neutrophils) (2). In this context, the phagosome environment is remarkably hostile toward internalized bodies, including pathogens, prompting rapid destruction and subsequent digestion. As such, during the maturation of the digestive vesicle, many proteins are directed to act in the phagosome. The vacuolar ATPase (v-ATPase) gradually lowers the organelle pH making it considerably acidic (pH 4–5). Meanwhile, the NADPH oxidase complex (NOX) produces oxygen reactive species, like hydrogen peroxide (H_2_O_2_) (3–6). Another strategy for eliminating the phagocytized foreign body is the nutritional immunity, which can be defined as the modulation of the phagosomal traffic of specific metallic ions. This strategy regulates the influx and efflux to the organelle of transition metals that tend to transit between different stages of oxidation with moderate stability (7). The limitation or overexposure to such metals, especially iron (Fe), manganese (Mn), copper (Cu), or zinc (Zn), can compromise the infecticity of an internalized pathogen (7).

Both iron and manganese are transition metals essential to maintaining life due to their participation in numerous biological processes, acting as cofactors to enzymes such as oxidoreductases, hydrolases, transferases, and isomerases (8). Therefore, in amoebas and macrophages, the first attack on bacterial viability is through exporting Fe^+2^ and Mn^+2^ from the phagolysosome to the cytoplasm (9–11). In addition to limiting essential metals, the nutritional immunity strategy also takes advantage of the toxic properties arising from copper and zinc: their accumulation in the phagolysosome can interact with reactive oxygen species (ROS), potentiating oxidative damage to the phagocytosed pathogen (9). Although nutritional immunity efficiently eliminates many internalized agents, some fungi, bacteria, and viruses developed escape strategies to evade it.

Viruses are considered non-living pathogens due to the absence of an independent metabolism and, thus, portraying an absolute dependence on host cells to multiply (12). As the host metabolism needs iron for numerous processes, the viral infective process indirectly depends on the cell’s metal ions as well. Such requirements stem from their need as co-factors to several host enzymes that play a role in viruses’ replication and assembly (13, 14). Thus, it is possible that viruses present mechanisms that modulate such metal ions concentration in the cell. An example of viral influence on metallic ions on the cell is the iron overload during hepatitis C virus’ replication: at the cellular level, an increase in the expression of transmembrane proteins responsible for iron transport (13) is observed. Iron also stands out in the pathology caused by the human immunodeficiency virus (HIV-1), as infection also promotes intracellular iron overload which, in turn, releases NF-κB, resulting in the transcription of the HIV-1 genome previously integrated into the host cell genome. Furthermore, there are proteins involved in the transcription of the viral genome that are only active upon iron overload (13, 15, 16). Considering the importance of metallic ions on virus infection and replication in human viruses, it is compelling that metallic ions influence in viral replication and that it should be investigated in other members of the virosphere. In the present work, we will use a member of the *Nucleocytoviricota* phylum, the mimiviruses, to understand the role of ions during the initial steps of their replication. Recently, it was shown by our group that metal ions have the unexpected effect of opening the stargate on TPV. Interestingly, hydrogen peroxide also resulted in this drastic morphological movement (17). Thus, it stands to reason that metal ions probably have a critical role in the replicative cycle of other GVs, mainly mimiviruses.

In general lines, giant viruses (GVs) can be characterized by their enormous capsid size varying between 0.25 and 2 µm and varied morphology (icosahedral, oval, or spherical) (reviewed in reference 18). They include members of the *Mimiviridae* family, such as Acanthamoeba polyphaga mimivirus (APMV), Samba virus (SMBV), Tupanvirus (TPV), and Antarctica virus (19–21). APMV and SMBV have a capsid ~500 nm in diameter, covered by a dense layer of fibrils except for a specific apex where the stargate is located (22–24). TPV comprises ~450 nm diameter capsids covered by fibrils and present a stargate in one of its vertices. However, TPVs differentiate from other GVs by possessing a tail located at the opposite capsid axis to the stargate. This cylindric tail varies considerably, generally having >500 nm in length and is also covered by fibrils (25, 26). Antarctica virus was isolated by our group and still requires genomic analysis. However, it shares morphological characteristics with other members of the *Mimiviridae* family (21).

The replication cycle of mimiviruses, in general, shares similar stages. First, GVs are internalized by professional phagocytic cells, such as amoebas, and are sequentially entrapped in phagosomes (18). Next, maturation of phagosomes through its fusion with the lysosome occurs, resulting in the formation of the phagolysosome. After about 1 h, the stargate opening is likely induced by intraphagosomal iron, cooper, and/or hydrogen peroxide (17), allowing the fusion between the viral and phagolysosome membranes (23, 27). These fusions form a passage through the phagolysosome to the cytoplasm and release both the viral genome and the viral seed. The latter is a vesicle-like structure that contains macromolecules required for the subsequent steps of the cycle (23, 27). Upon release of the genome into the cytoplasm, a pseudo-organelle known as the viral factory is formed within 3–6 h. This large structure occupies ~80% of the amoeba’s cytoplasm and is where transcription and translation of the viral genome occurs, as well as particle assembly. After ~72 h of infection, the amoeba’s cytoplasm is filled with viral progeny leading to cell lysis, and viral particles are released into the environment (23, 26, 28).

One of the first steps in mimivirus infection is characterized by the opening of the stargate. A previous study from our group observed that the capsid of APMV, SMBV, TPV, and Antarctica can be opened *in vitro* under extreme conditions such as acidic pH (<3), high temperatures (100°C), and high salt (~4M) (27). These findings showed that stargate opening is a multi-stage process and that mimivirus capsids are exceptionally stable. Thus, we followed on to pursue phagosomal components that could also induce capsid opening. Hydrogen peroxide, copper, and iron ions are also involved in stargate opening *in vitro* (17), all elements present in the phagosome maturation route, and part of the nutritional immunity defense strategy (9).

The importance of transition metals in the different replicative cycles of the most diverse pathogens is evident. Our pioneering work demonstrated that iron plays a crucial role in opening the stargate. Therefore, we followed on this path: here, we demonstrate that GVs have different sensitivities to metals, cellular uptake is increased during the opening of the stargate and that iron increases progeny production of APMV and TPV. Our results add information to the molecular puzzle presented by this unique viral family.

## MATERIALS AND METHODS

### Cell culture

Acanthamoeba castellanii trophozoites cell line (ATCC 30011) was grown in 75 cm^2^ cell culture flasks, maintained in 712 PYG with additives medium, prepared to a final volume of 1L [proteose peptone 20 g/L, yeast extract 1 g/L, 400 µM CaCl_2_, 4 mM MgSO_4_ × 7 H_2_O, 4 mM 2.5 mM Na_2_HPO_4_ × 7H_2_O, 2.5 mM KH_2_PO_4_, 50 µM Fe(NH_4_)_2_(SO_4_)_2_ 6H_2_O, 100 mM glucose], supplemented with 1 mg/mL Penicillin-Streptomycin (LGC, USA), 15 µg/mL Gentamicin (Thermo Fisher Scientific, USA), and stored in an incubator at 28°C.

### Purification of giant virus

Virus stocks were obtained through the infection of *Acanthamoeba castellanii* cells (ATCC strain 30011) and, after 96 h of infection, were purified as described in reference Oliveira et al., 2019. Viral titration by TCID50 was performed as described by Reed and Muench (29).

### Cryo-EM analysis

Giant virus particles for cryo-EM imaging were incubated in either 20 mM sodium phosphate buffer, pH 7.4, 20 mM sodium phosphate buffer, pH 7.4, with 10 mM FeCl_3_, or 20 mM sodium phosphate buffer, pH 7.4, with 10 mM CuCl_2_. The samples were allowed to equilibrate for 1 h at room temperature (~25°C) prior to imaging. Small (~5 µL) aliquots of treated virus particles were applied to R2/2 Quantifoil grids (Electron Microscopy Solutions) that had been plasma cleaned for 60 s in a Pelco easiGlow glow discharge unit. The samples were plunge frozen in liquid ethane using a Vitrobot Mark IV set to 4°C and 100% humidity using 4 s of blotting time per grid and a blot force of 1. Frozen-hydrated samples were stored, transferred, and imaged under liquid nitrogen temperatures. Cryo-EM experiments were performed at the RTSF Cryo-EM Core Facility at Michigan State University. Virus particles were imaged using a Talos Arctica operated at 200 keV, under low dose conditions controlled by EPU. Micrographs were recorded on a Ceta camera operating in linear mode. Micrographs were collected at nominal magnifications ranging 11 k, 13.5 k, and 17.5 k (6.03, 7.60, and 9.66 Å/pixel, respectively). The objective lens defocus settings ranged from 5 to 10 µm underfocus. Micrographs were collected for 4–15 s, resulting in a total dose of <60 e-/Å2 per sample. The percentage of open giant virus particles was determined as previously described (27). In brief, this was calculated by dividing the number of clearly opened particles by the number of total particles visualized via 2D cryo-EM.

### Iron susceptibility assays for cell viability testing

*A. castellanii* cells (4.0 × 10^4^ cells/well) were grown in 96-well plates with 200 µL of PYG medium supplemented with 0, 50, 100, 200, 300, 400, or 500 µM of FeCl_3_. After 36 h, 50 µL of supernatants from each culture was placed in a new 96-well plate with 50 µL of CytoTox 96 Non-Radioactive Cytotoxicity Assay. Measurements were performed according to manufacturer’s instructions. The reaction was kept protected from light at 37°C for 30 min, and the absorbance was measured at 490 nm in a SpectraMax 5 plate reader (Molecular Devices in San Jose, California, USA).

### Measurement of intracellular iron

The concentration of intracellular iron accumulated under different conditions was determined by a colorimetric assay based on the use of ferrozine (30). *A. castellanii* trophozoites (1.5 × 10^6^ cells) were cultivated in 25 cm^2^ cell culture flasks containing PYG medium until complete cell adhesion occurred. Then, the supernatants were removed, and the amoebas were infected with APMV, TPV, or Antarctica virus at a multiplicity of infection (MOI) of 1, in PYG medium supplemented with 0 µM, 100 µM, 400 µM, or 500 µM FeCl_3_. After 1 h, media was removed, and cells were incubated with 5 mL of PYG medium. After 1 or 3 h of infection, cells were collected, centrifuged at 800  × *g* for 10 min, and washed three times in PBS. Cells were lysed with 200 µL of 50 mM NaOH for 2 h. Then, 100 µL of 10 mM hydrochloric acid was added to 100 µL of the cell lysate; ionic Fe^2+^ bound to the intracellular structure was released by adding 100 µL of a mixture of 1.4 M HCl and 4.5% potassium permanganate (KMnO_4_) (vol:vol), following 60°C incubation for 2 h, in the absence of light. After cooling down to room temperature, 30 µL of the reagent for iron detection (6.5 mM ferrozine [Sigma], 6.5 mM neocuproin [Sigma], 2.5 M ammonium acetate [Sigma], and 1 M ascorbic acid [Sigma]) was added and samples were incubated for 30 min at room temperature. The absorbance of each reaction was measured at a wavelength of 550 nm in the SpectraMax 5 plate reader (Molecular Devices, San Jose, CA, USA). The concentration of Fe^2+^ was determined using a standard curve with known FeCl_3_ concentrations (0-75 µM). In addition, protein concentration of each lysed sample was determined by the Lowry method (31), using BSA as a standard for protein concentration determination.

### Iron reductase assay

The cell-impermeable chemical potassium ferricyanide (K_3_Fe(CN)_6_) was used to evaluate the cells' capacity to reduce extracellular Fe^3+^ to Fe^2+^. *A. castellanii* trophozoites (1.0 × 10^6^ cells) were cultivated in 25 cm^2^ cell culture flasks containing PYG medium until complete cell adhesion occurred. Then, the supernatants were removed, and amoebas were infected with APMV or TPV at an MOI of 1 in PYG medium. After 1 h, media was removed, and cells were incubated with 5 mL of fresh PYG medium. Infection proceeded for 30 min, 1 or 3 h, after which cells were collected by centrifugation (800 × *g* for 10 min), washed three times in PBS, and lysed by freeze-thaw cycles. Homogenates were then resuspended in 1 mL of Hank’s Balanced Salt Solution (HBSS) containing 1 mM K_3_Fe(CN)_6_. The change in absorbance at 420 nm was used to track the reduction of K_3_Fe(CN)_6_ from its Fe^3+^ to Fe^2+^ state. The homogenate was centrifuged at 10,000 × *g* for 5 min after 3 h, and the supernatant was then read using a SpectraMax 5 plate reader (Molecular Devices in San Jose, California, USA). The millimolar K_3_Fe(CN)_6_ values from the A420 measurements were converted using a standard curve of K_3_Fe(CN)_6_. Iron-reductase activity was calculated after subtracting nonspecific K_3_Fe(CN)_6_ reduction seen in the absence of cells.

### Amplex Red peroxidase assay

The production of H_2_O_2_ by the trophozoite cells was assayed by measuring the rate of H_2_O_2_ reduction to H_2_O, using the Amplex Red oxidation (Invitrogen) fluorometric method, as described in Dick et al. (32). Briefly, *A. castellanii* were cultivated in 25 cm^2^ cell culture flasks containing PYG medium until complete cell adhesion occurred. Then, the supernatants were removed, and the amoebae were infected with APMV or TPV at an MOI = 1 in PYG medium. After 1 h, the media from the bottle was removed, and the cells were incubated with 5 mL of fresh PYG medium. After 0, 30 min, 1, 3, 8, 12, 16, or 24 h post infection, samples were treated with 5 mM Tris-HCl (pH 7.4), 1.7 M Amplex Red (Invitrogen, Carlsbad, CA, USA), and 6.7 U/mL horseradish peroxidase (Sigma-Aldrich), for 30 min at room temperature. Fluorescence was measured at 563 and 587 nm for excitation and emission, respectively. H_2_O_2_ concentration was determined using a standard curve.

### Superoxide dismutase activity

Total SOD activity was determined based on SOD’s ability to prevent the reduction of nitro blue tetrazolium (NBT) by O_2_^−^ as described in Dick et al. (32). *A. castellanii* cells were cultivated in 25 cm^2^ cell culture flasks containing PYG medium. Then, supernatants were removed, and the adhered amoebas were infected with APMV or TPV at an MOI = 1 in PYG medium. After 1 h, media was removed, and cells were incubated with 5 mL of fresh PYG medium. After 30 min, 1 or 3 h post infection, trophozoite cells (1.0 × 10^6^ cells) were pelleted, disrupted by freeze-thaw, and then washed three times in cold PBS. The protein content of the whole homogenate was calculated using the Bradford technique (33). The homogenates were incubated in a reaction medium comprising 45 mM potassium phosphate buffer (pH 7.8), 6.5 mM EDTA, and 50 mM NBT in a final volume of 200 µL using known protein concentrations in the range of 10–50 µg. The reaction was triggered by the addition of 2 mM riboflavin. After 15 min protected from light exposure in a dark box, the absorbance at 560 nm was measured. SOD activity was calculated as the percentage of enzyme-blocking NBT that was reduced per protein.

### Statistical analysis

All experiments were performed with biological triplicates, with similar results obtained from at least three different cultures and infections. The values presented in all experiments represent the mean standard error (±). Differences between groups were analyzed using Student’s *t* test, and with one-way ANOVA to verify differences within the experimental group using Prism 7.0 software (GraphPad Software, San Diego, CA). Statistical significance was considered at *P* < 0.05.

## RESULTS

### Stargate opening in TPV, APMV, and SMBV can be triggered by both iron and copper ions, albeit with differing sensitivities to each metal

Little is known about the composition of the biomolecules that form the GVs virions. Recently, our group, using the Energy Dispersive X-ray (EDX) microanalysis technique, observed the binding of specific metal and non-metal components on TPV capsid (17). The study showed that CuCl_2_ and FeCl_3_, but not other tested metals, promoted stargate opening. These results offer new perspectives about how metal ions can modulate virus structure (17).

**Fig 1:**
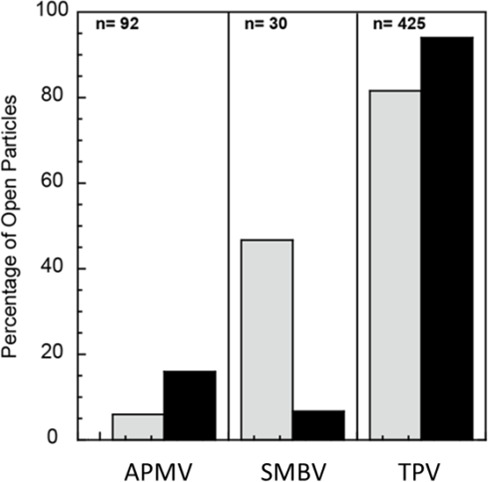
Iron and copper open the stargate structure present in the capsids of APMV, SMBV, and TPV. The virus particles were incubated in 20 mM sodium phosphate buffer at pH 7.4, with 10 mM FeCl₃, or 20 mM sodium phosphate buffer at pH 7.4, with 10 mM CuCl₂, for 1 h at room temperature. Subsequently, the samples were processed for analysis using cryo-EM images. The bar graph shows the percentages of APMV, SMBV, and TPV opened particles after incubation with FeCl_3_ or CuCl_2_. Gray bars represent the percentage of capsids opened after treatment with 10 mM FeCl₃, and black bars represent those opened after treatment with 10 mM CuCl₂.

Given our pioneering results, we decided to test the influence of iron and copper in Antarctica, APMV, SMBV stargate opening process, as both metals are abundantly present in the phagosome of *A. castellanii*, the typical host for these viruses. APMV had ~6% and ~16%, SMBV had ~47% and ~7%, TPV had ~82% open particles upon copper treatment and ~94% after iron treatment, respectively (Fig. 1). Notably, stargate opening efficiencies differ among the three mimiviruses. Thus, we hypothesize that although similar, they likely rely on distinct molecular mechanism to trigger stargate opening. Iron chloride consistently proved to be the most effective inducer of stargate opening. Based on this observation, we focused our investigation on the role of this metal during the early stages of the viral replication cycle. Our analysis centered on the first three hours post-phagocytosis, an interval closely associated with stargate opening.

### APMV and TPV increase iron uptake by amoebas during the first hours of infection

**Fig 2:**
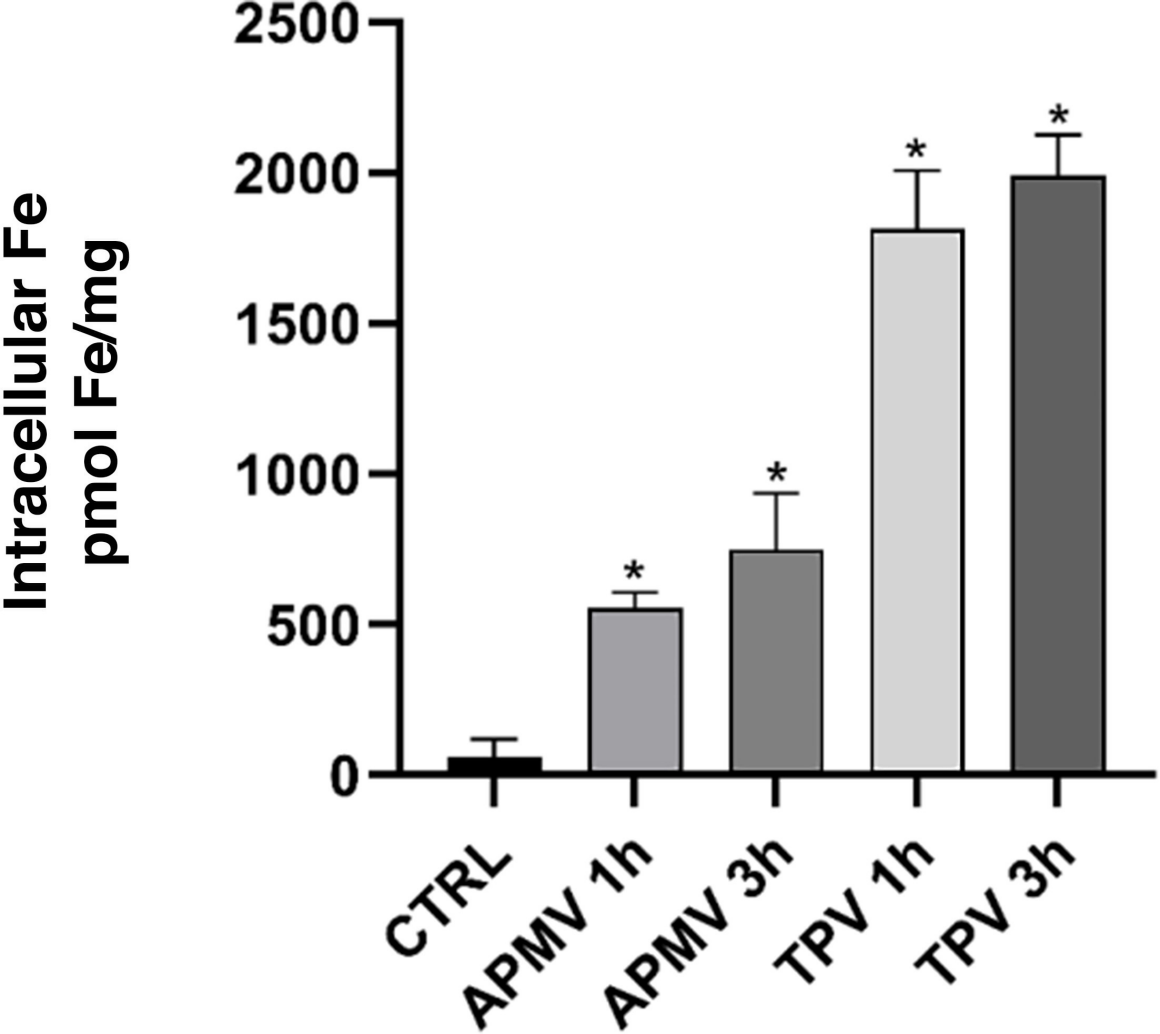
The intracellular iron content increases in the first hours of APMV and TPV infection. *A. castellanii* cells were infected with APMV, or TPV (MOI 1). After 1 or 3 h of infection, the intracellular iron concentration was measured. TPV showed the highest increase in metal concentration: in 1 h, iron was 9.5× or at 3 h, 10.5× higher than that of uninfected, control cells; in comparison, the intracellular iron concentration detected for APMV-infected amoebas was 7.3× and 8.7× after 1 or 3 h.p.i. *P < 0.05.

To evaluate the intracellular dynamics of iron in infected amoebae, *A. castellanii* were infected for 1 or 3 h with APMV and TPV. The amoebae were then collected and washed to remove excess iron and subsequently lysed. The intracellular iron quantification data after infection with APMV or TPV is shown (Fig. 2). Compared to the control, the cell groups infected with APMV and TPV had an increase in internalized Fe^2+^: 7.3× to 8.7× or 9.5× to 10.5× increase in intracellular Fe^2+^ at 1 and 3 h of infection, for APMV and TPV infections, respectively. Therefore, as observed for other viruses, APMV and TPV infection can stimulate the increase in cellular iron uptake, likely to ensure the availability of this metal during different stages of infection. The overlapping time intervals between the variation in iron concentration and the stargate opening step of the replication cycle indicate a possible association between the two events.

### Pretreatment of cells with FeCl_3_ increases iron uptake during GVs infection

**Fig 3:**
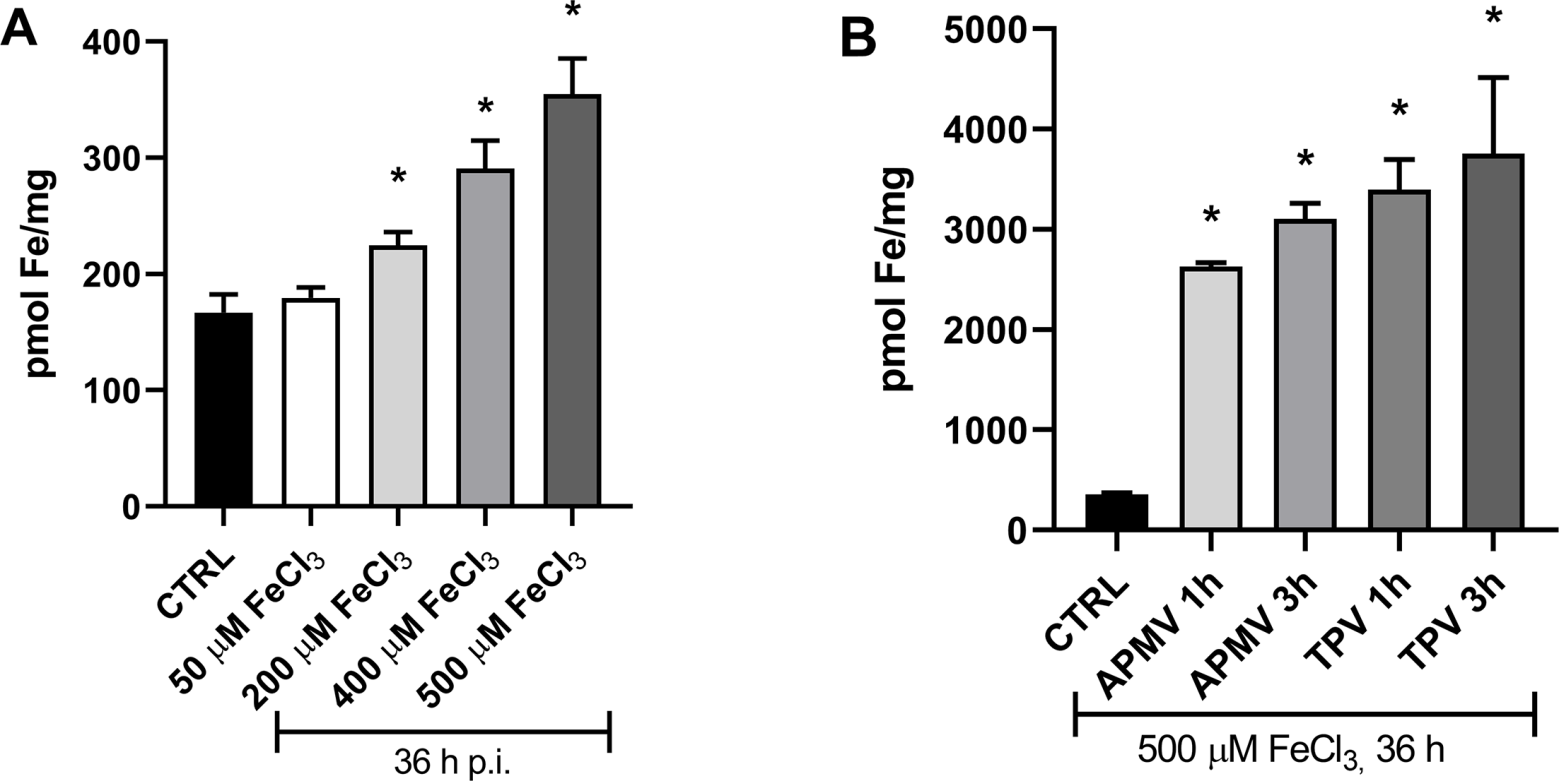
Effect of amoebal culture pre-treatment with different concentrations of FeCl_3_ on cell viability and iron uptake during APMV and TPV infection. (**A**) Intracellular iron content in *A. castellanii* cultured in PYG medium supplemented with increasing concentrations of FeCl₃. Cells (1 × 10⁶) were incubated for 36 h prior to iron quantification. (**B**) Intracellular iron content in APMV- or TPV-infected cells pre-treated with 500 µM FeCl₃ for 36 h. Following infection, cells were harvested and intracellular iron was measured. APMV infection resulted in a 7.3- to 8.7-fold increase in intracellular iron, while TPV infection led to a 9.5- to 11-fold increase. *P < 0.05.

To demonstrate that the APMV and TPV infections affect iron uptake by the cells, we analyzed the impact of an increase in iron availability and whether the amoebas would incorporate excess iron during mimivirus replication. Cells were incubated for 36 h with PYG medium was supplemented with 0, 50,200, 400, or 500 µM FeCl_3_. When compared to the control, intracellular iron concentration in infected cells increased 1.1× for 50 µM, 1.3× for 200 µM, 1.8× for 400 µM, 2.1× for 500 µM externally added iron chloride (Fig. 3A), with no detrimental effect on cell viability (data not shown). Thus, it is evident that the amoebas store more iron if its availability increases.

In a posterior experiment, cells were treated with 500 µM FeCl_3_ for 36 h. Subsequently, the supplemented media was removed, and cultures were washed with PBS to remove non-internalized iron. The cells were then infected with APMV or TPV at an MOI of 1. Intracellular iron concentrations were obtained at 1- and 3 h post-infection (Fig. 3B). Both APMV- and TPV-infected cells significantly increased intracellular iron concentration compared to values obtained in uninfected cells. APMV-infected cells presented an increase in iron concentration of ~7.3× and 8.7× at 1 h and at 3 h, respectively. Samples from TPV presented an increase of iron concentration of ~9.5× or ~11× at 1 or 3 h. These results suggest that APMV and TPV infections can increase the iron uptake in these cells.

### The presence of iron affects the viral titer

**Fig 4:**
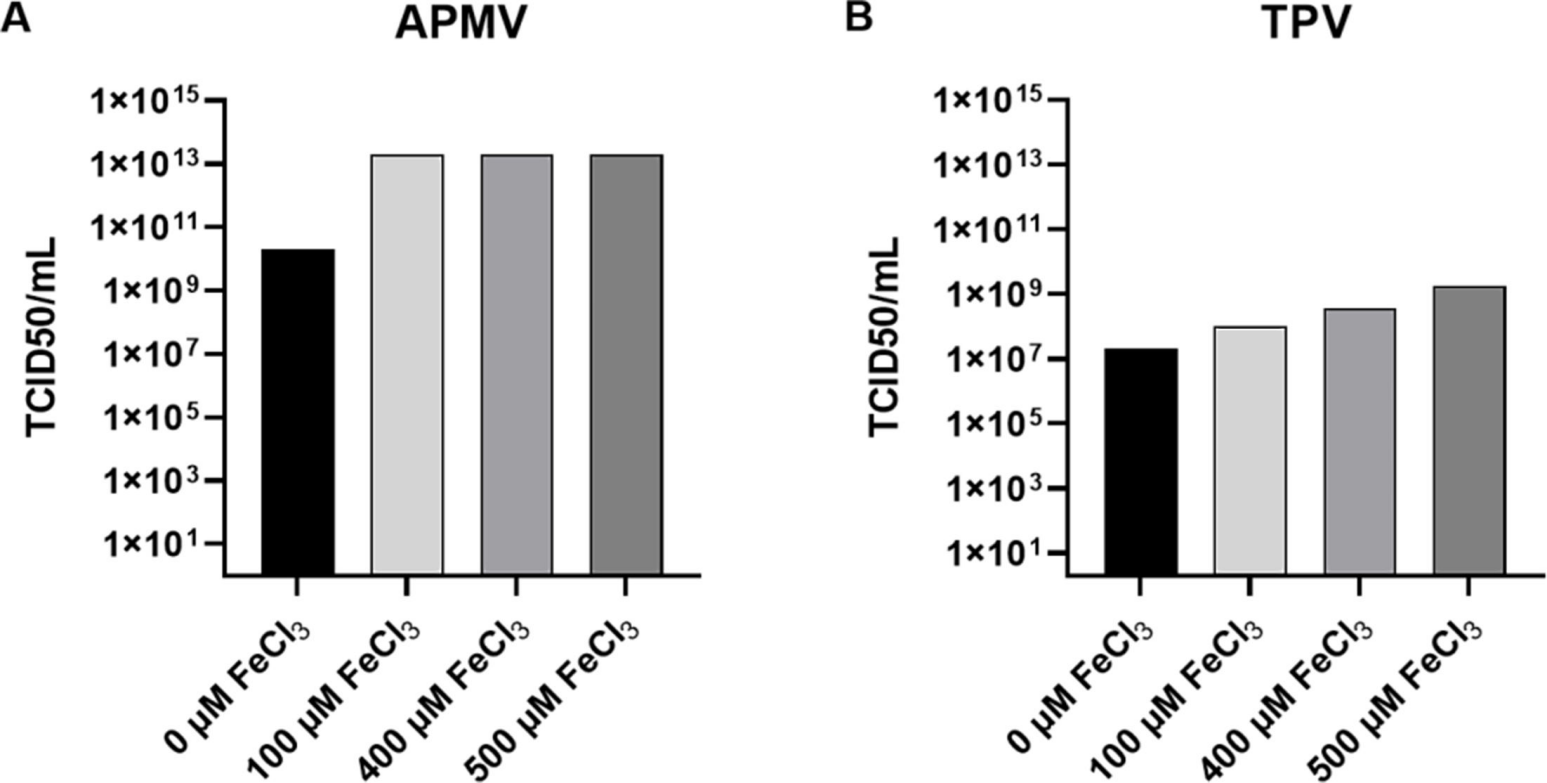
Effect of pretreatment with FeCl_3_ on the production of infectious APMV and TPV particles. *A. castellanii* cell cultures were maintained with PYG supplemented with 0 µM, 100 µM, 400 µM, or 500 µM FeCl_3_ for 36 h. Subsequently, cultures were infected with APMV or TPV for 1 h. Upon virus purification, we detected that (**A**) APMV titer reached 10^13^ and (**B**) TPV 10^10^, an increase of 1,000- or 100-fold when compared to viruses obtained from cultures not previously treated with iron chloride. Mimivirus tittering experiments are extremely reproducible, and therefore, the error is negligible between replicates.

The follow-up inquiry was whether the increase of intracellular iron concentration promoted by APMV and TPV infection affects virus titers. Cultures were treated with 0 µM, 100 µM, 400 µM, or 500 µM FeCl_3_ for 36 h and subsequently infected. As shown in Fig. 4A, the iron concentrations used increased the APMV titer by 1,000× compared to the control (10^13^ TCID50/mL vs 10^10^ TCID50/mL). For TPV, the pretreatment of cells with FeCl_3_ caused an incremental increase of up to 100× in the viral titter, as compared to the control (10^9^ TCID50/mL vs 10^7^ TCID50/mL, Fig. 4B). These results indicate that iron positively influences viral production, although the difference in viral titers probably occurred due to APMV having a lower incidence of misassembly particles than TPV, as discussed below.

### Infection with giant viruses increases iron reductase activity

Our data so far indicated that intracellular iron concentration increases as mimivirus infection proceeds, induced by the intracellular presence of APMV and TPV particles. So, we investigated whether host molecular elements could be involved in this phenomenon, more specifically AcFERED reductase, an iron reductase enzyme. Grechnikova et al. (34) performed a broad characterization study of *A. castellanii* proteins directly involved in iron metabolism (34). By moderately depriving the amoebae of iron, proteomic analysis demonstrated an upregulation of the reductase enzyme. AcFERED is homologous to metalloreductase 6-transmembrane epithelial antigen of the prostate (STEAP) and reduces Fe^3+^ to Fe^2+^. This transporter is responsible for transporting Fe^2+^ out of digestion vesicles that act as storage sites for the product of phagocytosis or pinocytosis (34).

**Fig 5:**
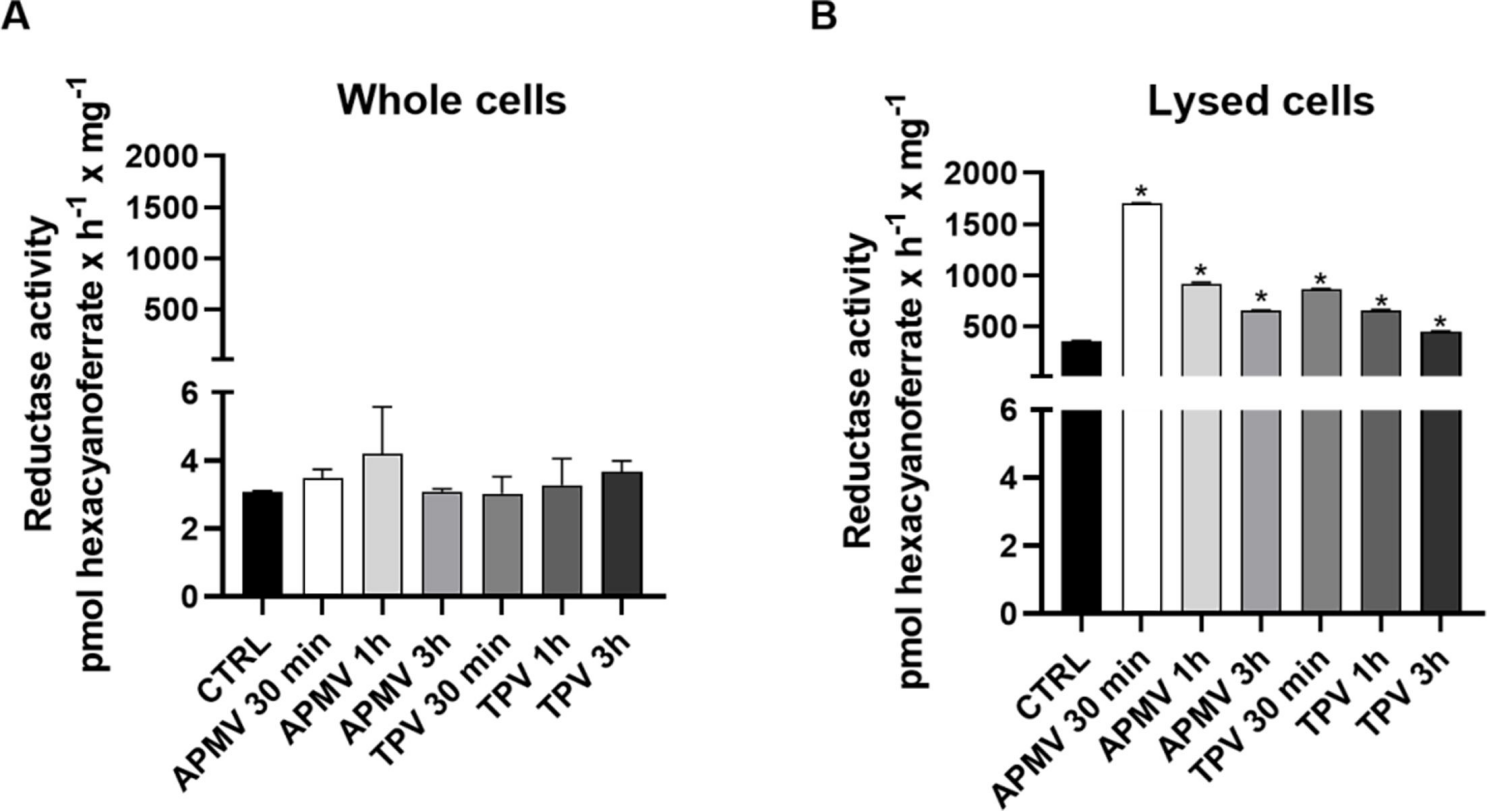
AcFERED reductase activity assay of *A. castellanii* infected with APMV or TPV. *A. castellanii* cells (1.0 × 10^6^ cells/mL) infected with APMV or TPV for 30 min, 1 or 3 h and further processed to obtain the Fe-reductase activity for the whole cell (**A**) or the cell lysate (**B**). Activity in the cell lysate was considerably higher likely due to the complete exposure of the intracellular compartments with higher AcFERED expression. *P < 0.05.

In order to test if AcFERED reductase could be associated with the observed increase of intracellular iron, the reductase activity was measured throughout GVs infection. Cell cultures were infected for 30 min, 1 and 3 h with APMV or TPV. The reductase activity was measured by the reduction of extracellular Fe^3+^ to Fe^2+^ using potassium ferricyanide (K_3_Fe(CN)_6_) as a tracker. Whole cells and the homogenate of lysed cells were used. Measurements performed on whole cells showed no significant changes in reductase activity (Fig. 5A), while the lysate showed increased activity at all tested times for both viruses (Fig. 5B). For APMV, the activity measured and its increase relative to the control was ~4.8× at 30 min, ~2.6× at 1 h, and ~1.8× at 3 h. For TPV, it was ~2.4× at 30 min, ~1.9× at 1 h, and ~1.3× at 3 h. We hypothesize that our lysed-cells assay showed a more significant difference due to AcFERED location, as compared to the whole-cells results. Measurements performed on lysed cells allowed access to reductases likely located in phagosomes as well. Thus, an increase in activity could be induced directly by the co-localization of the viruses and the reductase, which does not occur with whole-cell analyses.

### The presence of APMV and TPV in phagosomes alters the production of hydrogen peroxide and the activity of the enzyme superoxide dismutase

**Fig 6:**
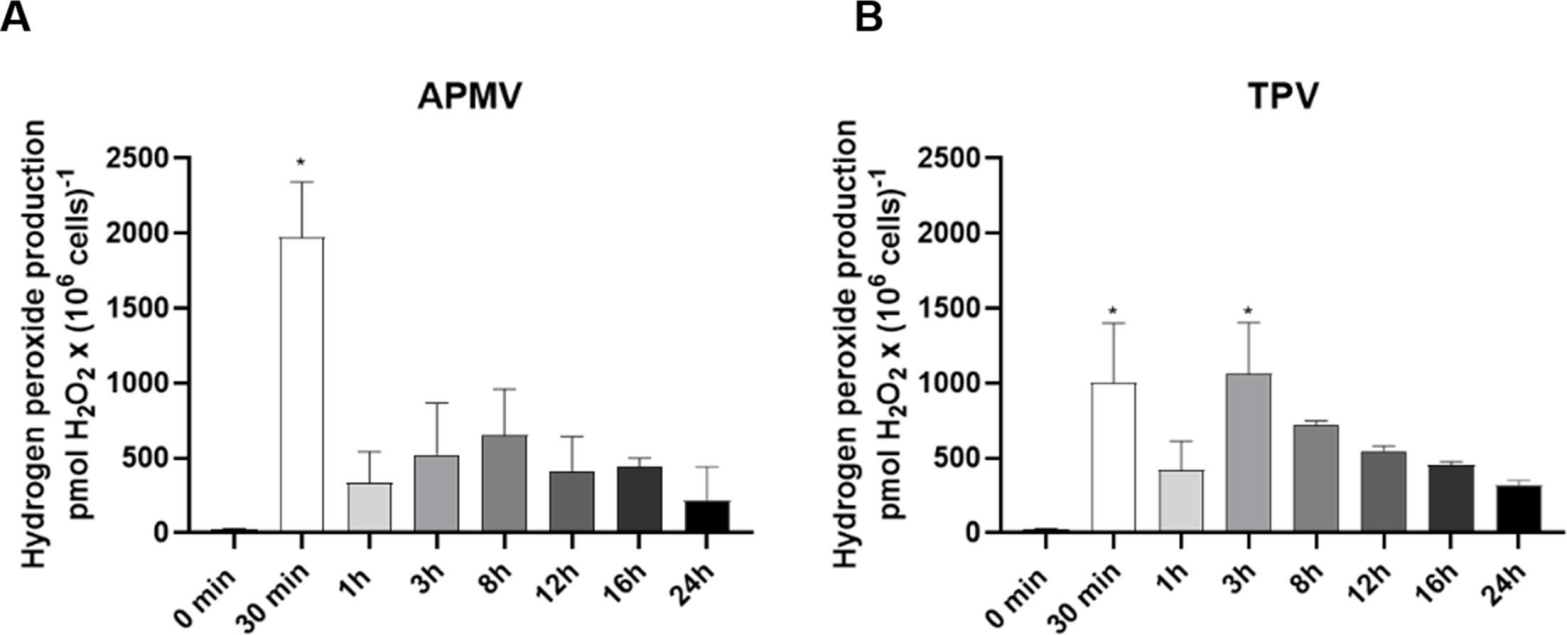
Variation of hydrogen peroxide production in amoeba during APMV or TPV infection. H_2_O_2_ production was evaluated at 0 min, 30 min, 1, 3, 8, 12, 16, and 24 h after infection with APMV (A) and TPV (B) using Amplex Red. *P < 0.05.

To complement the study of changes in the phagosomal environment generated by mimiviruses, we sought to measure the production of other components commonly present in them, such as hydrogen peroxide. Phagolysosomes are generated after the fusion of phagosomes with lysosomes. These vacuoles contain NADPH oxidase, which, in turn, produces reactive oxygen species (ROS) like H_2_O_2_. Allied with the strategy of nutritional immunity and pH drop, the controlled accumulation of H_2_O_2_ helps destroy pathogens trapped in phagolysosomes (35). Therefore, the production of H_2_O_2_ and the activity of SOD at different times of infection were measured. Production of H_2_O_2_ was analyzed at 0, 30 min, 1, 3, 8, 12, and 24 h post-infection for APMV and TPV. As expected, the amounts of H_2_O_2_ decline as the infection progresses (Fig. 6). APMV infection generates H_2_O_2_ production peaks at 30 min and 8 h, while TPV at 1 and 3 h. These differences in late peak peroxide production are likely due to the infection kinetics; previous data from our group showed that TPV takes approximately half the time to complete its replication cycle as compared to APMV (36).

**Fig 7:**
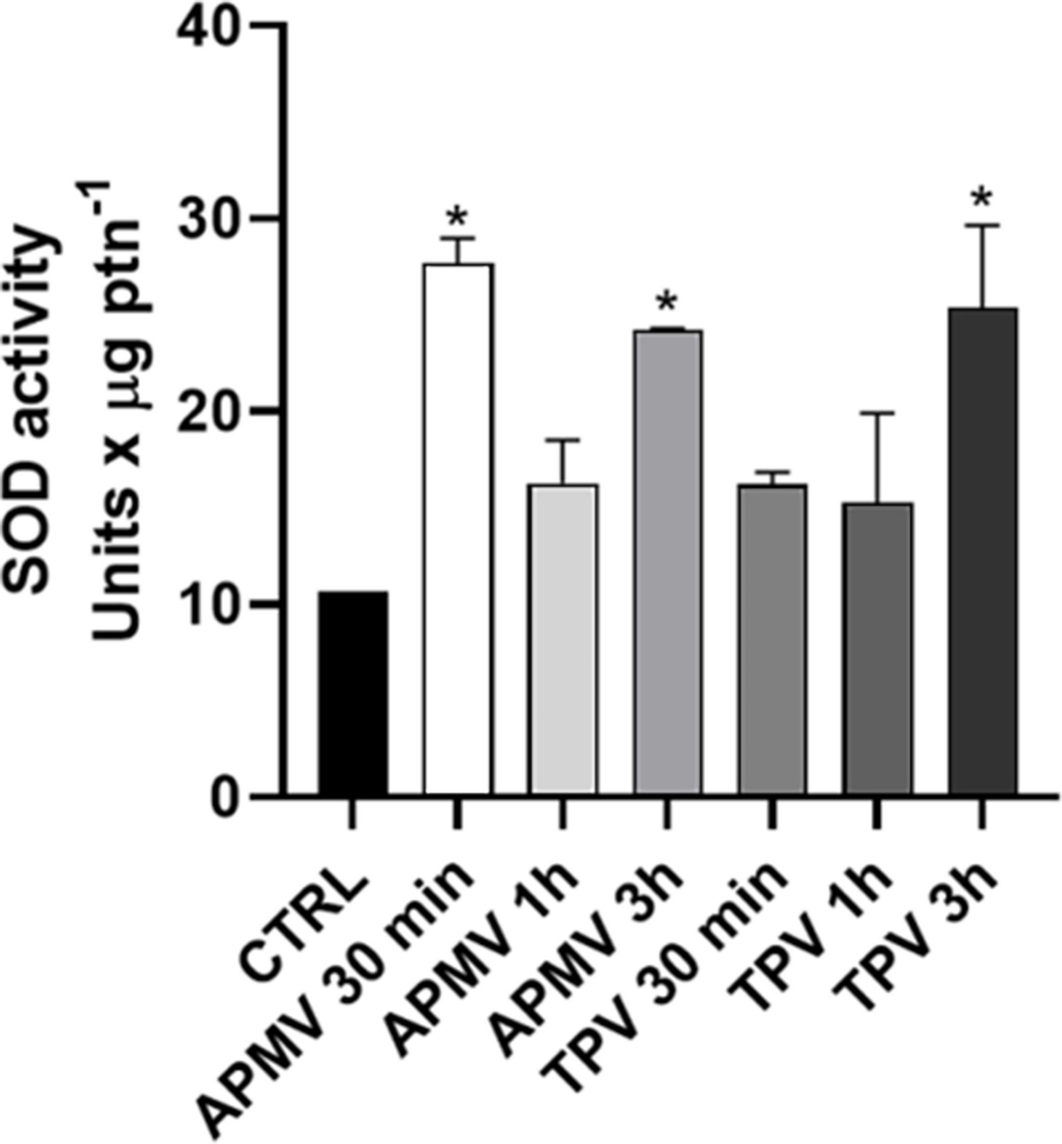
APMV or TPV infection increases the superoxide dismutase enzyme activity in amoebae. Cultures of *A. castellanii* (1.0 × 10^6^ cells) were infected for 1 h with APMV or TPV. Infected cells were collected 30 min, 1 or 3 hpi and centrifuged by 8,000 × *g* for 10 min. The supernatant was discarded, and the pellet was resuspended in 1 mL PBS and SOD activity was measured. A significant increase in SOD activity was detected at 30 min and 3 h in APMV-infected cells or at 30 min and 3 h for TPV-infected cells, as compared to non-infected cells. *P < 0.05.

In parallel, we observed a peak of SOD enzyme activity at 30 min and 3 h for APMV and at 3 h for TPV (Fig. 7), corroborating the infection-induced H_2_O_2_ production.

## DISCUSSION

Iron is a mandatory element in several cellular processes and is, therefore, required in viral infective pathways. In the context of mimiviruses, amoebas are cells that complement the requirements imposed by their replicative cycles. Phagocytosis allows the internalization of the entire viral particle, and at this point, the stargate must open so that the host cellular machinery can adequately access the genome (28). The stargate opening plays a critical role in the infection. Schrad and colleagues demonstrated that it is possible to trigger the SMBV and TPV stargate opening through pH acidification (below pH 4) (27). Although the pH of eukaryote phagolysosomes peaks at 4.5, certain species of amoeba vacuoles may be more acidic. *Dictyostelium amoebae*, a host for TPV, modifies the flow of metals after phagocytosis, forms phagolysosomes with pH values below 3.5 (25, 37, 38). We cannot rule out the possibility that multiple phagolysosome components, such as metals, peroxide, pH, and potentially others, may act synergistically to enhance stargate opening (17). We showed *in vitro* that iron and copper promote the stargate opening in APMV, SMBV, and TPV (Fig. 1). TPV was the most susceptible to the treatment, with the most particles with the stargate open, followed by SMBV and APMV. The variation in opening behavior between the viruses suggests a possible structural, biomolecular composition or stability difference in the stargate region amongst the tested viruses.

Through a comparative analysis, we demonstrated that cells infected by APMV or TPV for 1 or 3 h showed elevated levels of intracellular iron compared to the control (Fig. 2). It is also interesting to point out that the analyzed time points coincide with the period of the estimated permanence of the virus in the phagosome and stargate opening. Further supporting data showed that pretreating host cells with 500 µM FeCl_3_ before infections significantly increased intracellular iron concentration in mimivirus-infected cells (Fig. 3B), where TPV showed the highest mean increase in Fe^2+^ uptake (~10-fold), followed by APMV (~8-fold). Interestingly, TPV was more amenable to iron chloride in stargate opening. Maybe this could correlate with higher iron dependency than its related mimivirus. Further experiments will be required to describe the molecular explanation for such intracellular concentration peak differences in each of the replication cycles assayed.

We also analyzed whether the additional presence of iron would affect the viral progeny. At this point, once iron facilitates the opening of the stargate, the viral genome can be freed into the host cytoplasm for continued replication. Thus, we spiked the cultures with different iron concentrations for 36 h and then infected them with APMV or TPV. To corroborate our hypothesis, a significant increase in the viral titer of APMV and TPV was observed (Fig. 4). Interestingly, although APMV stargate opening has a lower metal susceptibility, its titer increased 1,000× upon 100 µM FeCl_3_ treatment. In turn, the TPV titer increased 100× at the highest iron concentration. We hypothesize this difference observed in viral titer between APMV and TPV (Fig. 4A and B) could be explained by a greater number of malformed viral particles in TPV than in APMV, especially because during virus expansion in the laboratory both viruses present a significant difference in yield. As TPV virion presents a complex structure with a large tail, there has been a reported great number of incomplete particles, such as empty or tailless capsid, after cell lysis when compared to other giant viruses (25, 39). Therefore, although TPV has a higher number of capsid opening during infection, it would have a lower number of viable particles at the end of the cycle. This lower number of infective particles is then reflected on the lower titer when compared to APMV (Fig. 4). Other factors could also be affecting the final viral count, but another method would be required to more precisely ascertain the cause, as even the literature is severely lacking in such area. Interestingly, the iron chloride-induced increase in titer can potentially be very valuable for future prospection experiments by expanding the chance of isolating low titer giant viruses.

Transport proteins (NRAMP1 homologs) and iron reductases were recently described to be present in amoebal phagolysosomes (34). To understand the interplay between mimivirus infection and intracellular iron concentration modulation, the activity of reductases in whole- and lysed-infected cells was measured (Fig. 5). In whole cells, access to reductases located in the phagolysosome was hindered. Therefore, we did not see alterations in relation to the control (Fig. 5A). In the lysed samples, however, an increase in activity was detected in early times of infection caused by APMV or TPV (Fig. 5B). Unfortunately, we were not able to evaluate how the reduced iron would be trapped in the phagolysosome so as not to be exported by NRAMP1. In this sense, we hypothesize other factors may be influencing the intra-phagosomal concentration of iron, i.e., possibly inhibiting this metal’s export by NRAMP1 or some unknown viral mechanism can act by inhibiting this transporter.

It was shown that hydrogen peroxide production increased at different hours post infection (Fig. 6). We believe that certain levels of H_2_O_2_ are required during infection by these two viruses.

Increase of hydrogen peroxide production in amoeba correlates with key steps during APMV and TPV infections. Interestingly, an “early” and “late” peaks were detected for both viruses: 30 min and 8 h for APMV and 30 min and 3 h for TPV. Early time points are possibly linked to stargate opening. Previous work from our group showed that hydrogen peroxide alone can induce stargate opening in Tupanvirs (17). Later time points are likely linked to early genome processing and steps leading to progeny formation, like protein translation, for example (40). Also, early viral factories have been observed as early as 2–4 h (41), correlating well with a smaller peak of ROS production detected at 3 h for TPV. The viral factory is the only site of genomic replication and assembly of viral particles. Certain viral infections are associated with increased production of ROS. For example, infection caused by Influenza virus A (IAV) triggers a substantial increase in ROS production. Recent studies have demonstrated that the high production of ROS in endosomes via NOX_2_ activity could block the antiviral response of primary murine macrophages (42, 43). Cells that utilize oxygen in their respiratory chain have an excellent ROS detoxification system. This system includes SOD and catalase. SOD is an enzyme that acts in the reduction of O_2_^−^ into O_2_ and H_2_O_2_, and catalase acts to transform H_2_O_2_ into H₂O + O_2_. For IAV, an inhibition of SOD activity was noted so that O_2_^−^ levels were maintained and viral replication was favored (44). Our data showed high SOD activity in cells infected by APMV or TPV (Fig. 7), which can possibly be a cellular response to manage oxidative stress associated with viral morphogenesis. These findings align with recent evidence showing that intracellular pathogens such as *Legionella pneumophila* can subvert the antioxidant defenses of *Acanthamoeba castellanii* to favor their replication (45). This highlights a broader strategy among amoeba-infecting microorganisms to manipulate the host redox environment. Moreover, the modulation of oxidative stress during infection is not restricted to one class of viruses, as SARS-CoV-2, HIV, and Zika Virus pathogenesis are directly affected by the redox state of the host cell, for example. A recent review also underscored the multifaceted role of oxidative stress in both facilitating and restricting various viral infections, depending on the virus and host context (46). But understanding the full scope of how redox events affect viral infections still requires continued, deep investigation of different viral models.

The present results broaden the horizon for further studies about not only the peculiarities of the interaction between mimiviruses and their amoebal hosts but also the molecular mechanisms regulated by metal ions during their replicative cycle.

### Conclusions

**Fig 8:**
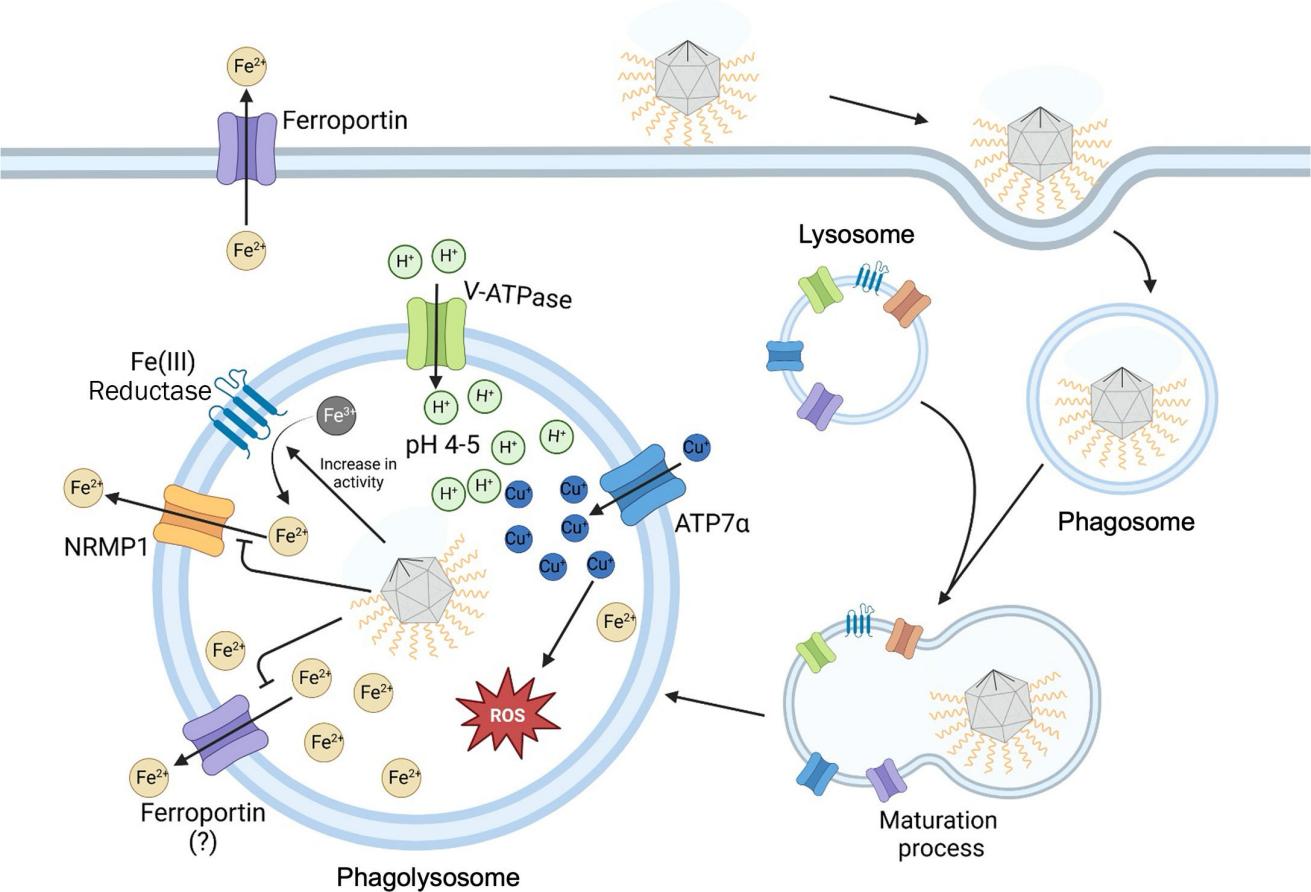
Schematics of viral uptake and interaction with ions in the phagolysosome. Illustration representing the process of viral uptake by the amoeba, its entrapment in the phagosome, the subsequent maturation process, and the interaction between the virus and ions present in the mature phagolysosome. The maturation process consists of the fusion/fission events between the lysosome and the phagosome, resulting in the mature digestive vesicle. In the resulting phagolysosome it is theorized that the entrapped virus influences ionic traffic. The Fe^2+^ ions are imprisoned into the vesicle due to the increase of iron-reductase activity together with NRAMP1 and ferroportin inhibition. Copper uptake into the vesicle is promoted by the cell and is believed it can play a role in stargate opening. Viral influence likely supports ROS production to promote the capsid opening.

Our results strongly suggest that iron ions may play an essential role during APMV and TPV infection, especially during the capsid opening step (Fig. 8). Based on the data presented, we hypothesize that the presence of APMV or TPV halts iron ions export from phagosomes, as well as induces the increase in the activity of iron reductases, by unknown mechanisms. In parallel, ROS production is tolerated up to 3 h after infection Interestingly, ROS is likely required for mimivirus replication. These apparent strategies adopted in early steps of GV infection are rather unique and reinforce the peculiarity of the interaction between different GVs and their hosts.

## Data Availability

All data and associated code are available within the paper and its supporting information files.
